# Validation of the Persian Translation of the Children’s Test Anxiety Scale: A Multidimensional Rasch Model Analysis

**DOI:** 10.3390/ejihpe10010006

**Published:** 2019-08-02

**Authors:** Roya Shoahosseini, Purya Baghaei

**Affiliations:** English Department, Islamic Azad University, Mashhad Branch, Mashhad 9187147578, Iran

**Keywords:** test anxiety, validation, children’s test anxiety scale, Rasch model, rating scale model

## Abstract

In this study, we examine the psychometric properties of the Persian translation of the Children’s Test Anxiety Scale (CTAS) using the Rasch rating scale model. In the first step, rating scale diagnostics revealed that the thresholds were disordered. To remedy this problem, two categories were collapsed and a rating scale structure with three points turned out to have optimal properties. Principal component analysis (PCA) of standardized residuals showed that the scale is not unidimensional. Since the scale is designed to measure three distinct dimensions of test anxiety we fitted a correlated three-dimensional Rasch model. A likelihood ratio test showed that the three-dimensional model fits significantly better than a unidimensional model. Principal component analysis of standardized residuals indicated that three subscales are unidimensional. Infit and outfit statistics indicated that one item misfitted the model in all the analyses. Medium correlations between the dimensions was evidence of the distinctness of the subscales and justifiability of the multidimensional structure for the scale. Criterion-related evidence was provided by correlating the scale with the Spence Children’s Anxiety Scale (SCAS). The patterns of correlations provided evidence of convergent-discriminant validity. Findings suggest that a three-dimensional instrument with a 3-point Likert scale works best in the Persian language.

## 1. Introduction

Tests are commonly given to school-aged children to make important decisions about their progress and status. In other circumstances, for accountability reasons, schools are mandated to administer tests to monitor students’ achievement across the teaching /learning cycle. This increased testing has had somewhat counterproductive impacts on students’ lives and achievement. Tests lead to anxiety which in turn has negative effects on students’ learning, academic performance and grades, and can also lead to drop out (Beidel and Turner, 1988 [1]; Chapell, et al., 2005 [2]; Tobias, 1979 [3]). The impact of anxiety on test performance can also lead to test validity concerns (Wren and Benson, 2004 [4]). If test scores are unduly affected by factors other than the construct of interest then the meaningfulness and usefulness of the scores are questioned. 

Test anxiety is defined as feelings of tension, fear, and worry experienced by students in evaluative situations (Spielberger and Vagg, 1995 [5]). It is generally agreed that the test anxiety construct is complex and multidimensional (Zeidner, 1998 [6]). There are several test anxiety models (with varying perspectives) that incorporate different aspects of human behavior into the construct model. While early models viewed test anxiety as general anxiety in evaluative contexts the modern views regard it as a construct with strong foundations in cognitive/attentional and personality domains (for an overview see Lowe, et al., 2008 [7]; Zeidner, 1998 [6]). 

Test anxiety is a prevalent phenomenon among students. Research indicates that between 25% to 40% of students experience some form of test anxiety, with minorities and females experiencing higher levels. The rate of the prevalence might increase as a result of increased testing in schools which could eventually lead to severer academic failures among students (Baghaei and Cassady, 2014 [8]).

The application of anxiety scales developed for adults may not be justified when used for children as their validity and reliability evidence was gathered in adult populations (Wren and Benson, 2004 [4]). Wren and Benson (2004) [4] argue that due to developmental differences the construct of test anxiety is not the same in children and adults and child exam anxiety measures should be based on a theory of exam anxiety for children.

Due to the unavailability of a modern exam anxiety measure for children in Persian we translated and validated the Children’s Test Anxiety Scale (CTAS) scale in Persian. The CTAS was selected for translation and adaptation in Persian because it is developed for application in a multiethnic population and was validated with such samples. Therefore, the scale seemed to be an appropriate candidate for translation and adaptation in other languages and cultures as its focus is on a diverse multiethnic student population.

## 2. Children’s Test Anxiety Scale (CTAS)

The Test Anxiety Scale for Children (TASC; Sarason et al., 1960 [9]) was the main instrument for measuring anxiety for almost four decades. TASC is a self-report instrument designed for children in grades 1–6, and contains 30 yes/no items. The TASC’s validity and usefulness came under attack in the early 2000s. Wren and Benson (2004) [4] questioned the validity and applicability of the instrument for the following three reasons: “outdated and/or overly complex wording of some items, outdated domain definition, and dimensionality issues” (p. 228). Wren and Benson argued that some of the items on the TASC are too long and wordy and some items are obsolete because of the changes in the schooling systems. The more serious criticism of the TASC relates to its outdated construct theory, as it does not take into consideration the more recent aspects of test anxiety, and its dimensionality. The TASC was developed as a one-dimensional measure. However, early research demonstrated that the scale is multidimensional (Dunn, 1964 [10]; Feld and Lewis, 1967 [11]). This multifactorial structure was later confirmed by other researchers too (Ludlow and Guida, 1991 [12]; Rhine and Spaner, 1973 [13]). Furthermore, the TASC had been introduced at a time when minorities and ethnic groups were not represented in American schools and the measure mostly addressed the white American population. 

To remedy the problems of the TASC, Wren and Benson (2004) [4] developed and validated the multidimensional Children’s Test Anxiety Scale (CTAS). The scale was developed based on the recent literature on models of child test anxiety and interviews with children about their feelings concerning exams. The test was designed for children aged 8 to 12. The CTAS is based on the theoretical assumption that exam anxiety in children has cognitive, somatic, and test-irrelevant behaviour symptoms (for more details see Wren and Benson, 2004 [4]). The test contains three subscales of Thoughts (13 items), Autonomic Reactions (9 items), and Off-Task Behavior (8 items). The Thoughts component includes the test-relevant and test-irrelevant concerns like self-deprecating ruminations and worries about the test’s impact which occur during testing. Autonomic Reactions pertains to the physiological and somatic reactions such as elevated heart rate during testing. Off-Task Behavior refers to the nervous habits and distracting behaviors like playing with cloths and biting the pencil during tests. Using confirmatory factor analysis, Wren and Benson (2004) [4] demonstrated the feasibility of their postulated three factor model. The scale is self-report questionnaire with items on a 4-point Liker scale (*almost never* = 0, *some of the time* = 1, *most of the time* = 2, *almost always* = 3). None of the items require revers scoring. The score on each subscale is the sum of the scores on the individual items. 

## 3. Method

### 3.1. Translation Procedure

The scale was translated from English into Persian (Farsi) by the researchers with a focus on transliteral equivalence. Great care was taken to select vocabularies and structures which are appropriate for elementary school children. The translated scale was reviewed by two other bilingual colleagues. The suggested changes were implemented where deemed essential by a panel of bilingual educationalists. The final version of the Persian scale was back-translated into English by another bilingual colleague. The original and the back-translated scales were contrasted to detect any possible discrepancies or misinterpretations. No such instances were detected.

### 3.2. Participants 

The Persian Children’s Test Anxiety Scale (PCTAS) was given to 160 Persian-speaking school children (89 girls) aged 8 to 14 in Iran (M_age_ = 12.88, standard deviation (SD) = 1.96) in their regular class time. Out of the 160 participants, 24% were 8–10 years old, 29% were 11–12 years old, and 47% of them were above 12 at the time of testing. Like the original scale, the PCTAS was scored on a 4-point Likert scale (*almost never* = 0, *some of the time* = 1, *most of the time* = 2, *almost always* = 3). 

Along with the PCTAS, the Spence Children’s Anxiety Scale (SCAS, Spence, 1998 [14]) was also given. The SCAS was selected as a criterion and the correlations between the PTASC and its subscales and SCAS and its subscales were computed. SCAS is a 44-item self-report questionnaire which measures general and specific anxiety disorders in children. The scale consists of six subscales of *Separation Anxiety Disorder*, *Social Phobia*, *Obsessive-Compulsory Disorder*, *Panic Attacks and Agoraphobia*, *Physical Injury Fears*, and *Generalized Anxiety Disorder*. Spence (1998) [14] reports validity and reliability evidence for the SCAS. We used the Persian translation of the scale validated by Anisi (2008) [15]. We selected this scale because to our knowledge it is the only validated scale for measuring general and specific anxiety in children in the Persian language. The Cronbach’s alpha for the total scale scores was 0.81 in this study. The Cronbach’s alpha reliabilities of the subscales are reported in Table 5. All subjects gave their informed consent for inclusion before they participated in the study. The study was conducted in accordance with the Declaration of Helsinki, and the protocol was approved by the Ethics Committee of Islamic Azad University, Mashhad Branch (institutional review board decision no. d/312).

### 3.3. Data Analysis

The data were analysed with the unidimensional Rasch rating scale model (Andrich, 1978 [16]) using the ConQuest software programme (Wu, Adams, and Haldane, 2007 [17]). Our sample size (*n* = 160) is large enough for Rasch model analysis (Linacre, 1994 [18]). Fit of data to an items response theory model like the Rasch model (Rasch, 1960/1980 [19]) is evidence that the covariation among the items can be explained with a latent dimension and all the items deepened on this dimension without sharing additional variance due to other sources (Baghaei and Shoahosseini, 2019 [20]; Baghaei and Tabatabaei Yazdi, 2016 [21]). In other words, inferences from test scores to a latent ability which explains consistency in item responses can be made. That is, the trait accounts for the items responses and the performances. This kind of evidence is at the heart of validity (Borboom, Mellenberg, and van Heerden, 2004 [22]).

## 4. Findings

### 4.1. Rating Scale Structure

The rating scale diagnostics revealed that the threshold parameters were disordered with the initial scoring of 0123. Thresholds are points on the latent continuum where the probability of endorsing a category and the adjacent category is the same (Linacre, 2017a [23]). Threshold estimates show the distinctiveness of each step on a Likert scale. They also show the sufficiency of the number of categories. The thresholds should neither be too close nor too far from each other (Bond and Fox, 2007 [24]). To remedy the problem of disordered thresholds, the categories were collapsed and two new scoring systems were tried. We collapsed the scores as 0122 and 0112. 

As Table 1 shows, in first scoring (0112) the thresholds become ordered but the distance between them is very small (−0.18 and 0.18). Linacre (1999) [25] recommends that the distance between thresholds should be at least 1.4 logits to indicate the distinctiveness of the steps and no more than 5 logits to avoid loss of information because of lack of enough distinguishable categories. In the second scoring (0112) the difference between the two thresholds was satisfactory (−1.01 and 1.01). Therefore, the scoring scheme 0112, i.e., a response scale with three points, leads to a better functioning rating scale. This scoring procedure was selected as the final scoring strategy and the basis of further analyses.

### 4.2. Unidimensionality Analysis

We checked the unidimensionality of the test using principal component analysis (PCA) of standardized residuals (Smith, 2002 [26]) with Winsteps Rasch programme (Linacre, 2017b [27]). Residuals are part of the data the model has not explained, so we expect them to be uncorrelated and randomly distributed (Linacre, 2017a [23]). To determine whether the data are unidimensional residuals are subjected to PCA. If the test is unidimensional no factor should be extracted from the residuals. To evaluate if the factors extracted from the residuals are ignorable or not, the size of their eigenvalues are examined. The size of the eigenvalue in the first factor is a measure of unidimensionality or global fit of data to the Rasch model (Smith, 2002 [26]). Eigenvalues above 2 suggest that the dimension extracted from the residuals is above noise level and is a secondary dimension that threatens the unidimensionality of the scale and is evidence that the data do not fit the Rasch model (Linacre, 2017a [23]). PCA of standardized residuals for these data showed that there are 2 contrasts or components with eigenvalues above 2. The eigenvalue for the first contrast was 3.0, for the second contrast was 2.3, and for the third contrast was 1.9 which was less than 2 so it did not illustrate any new dimension.

As there were two contrasts whose eigenvalues were greater than 2, this showed that there are secondary and tertiary dimensions in the items and the unidimensional Rasch model does not fit the data as indicated by the eigenvalue of the first and second contrasts in the residuals. So we used the multidimensional Rasch model to accommodate the different dimensions at the same time. 

The multidimensional random coefficient multinomial logit model (Adams, Wilson, and Wang, 1997 [28]) as implemented in ConQuest (Wu, Adams, and Haldane, 2007 [17]) was employed to estimate the model parameters. We tested a three-dimensional model, where three dimensions of Thoughts, Off-Task Behavior, and Autonomic Reactions were modeled to be three separate and correlated dimensions. The likelihood ratio test demonstrated that three-dimensional model fits significantly better that the unidimensional model. The difference in G^2^ (−2 loglikelihood) of the models is chi square distributed with degrees of freedom equal to the difference between the number of parameters (Briggs and Wilson, 2003 [29]), χ^2^ = 131.67, *df* = 5, *p* = 0.00. 

Table 1 shows the information criteria, reliabilities, threshold estimates, and other properties for the estimated models. The four models were also compared with the information criteria of AIC, (Akaike’s information criterion), BIC (Bayesian inference criterion), and CAIC (consistent Akaike’s information criterion). The model with smaller information criteria is better and is selected as a better fitting model. Information criteria showed that the three-dimensional model and the scoring 0112 have the best fit. Table 1 also depicts that the distance between the thresholds is slightly greater in the three-dimensional model. 

**Figure 1:**
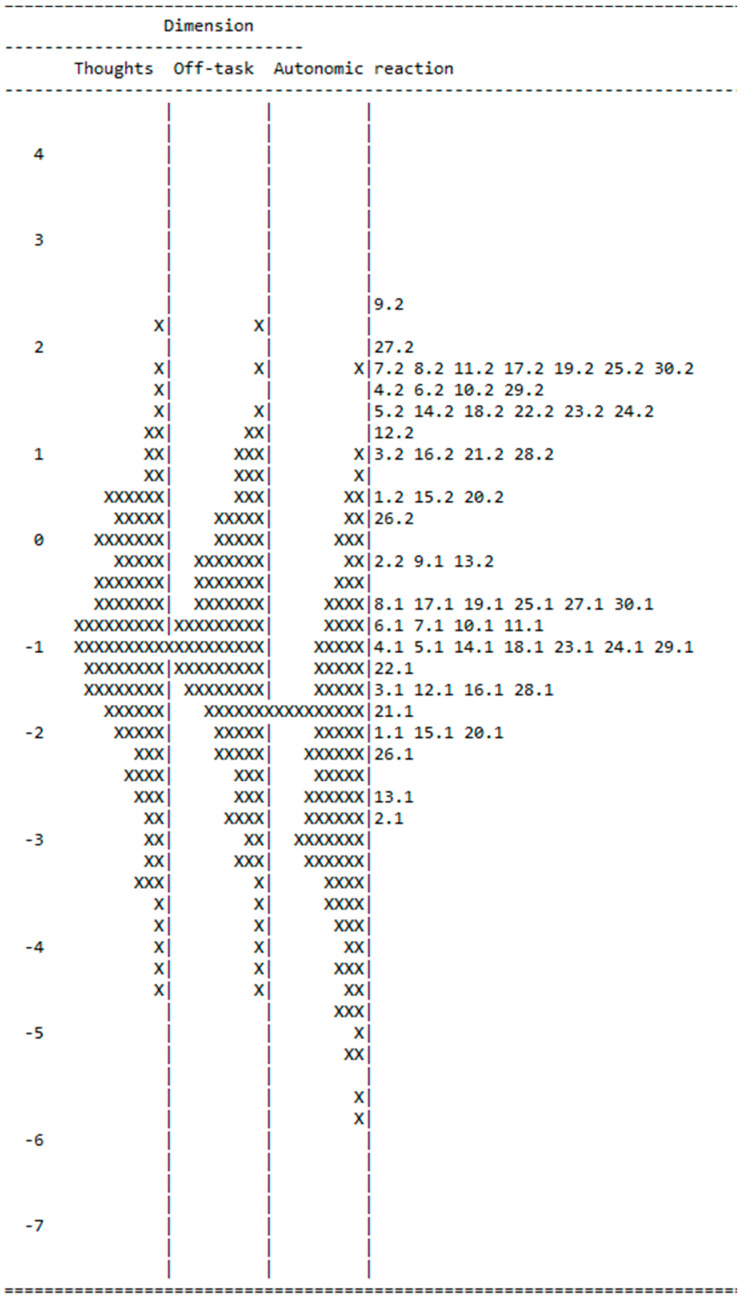
Map of latent distribution for the three dimensions.

Table 2 presents the parameter estimates for the 30 items, their standard errors, and their outfit and infit mean square statistics (MNSQ) in the multidimensional model. Infit and outfit values flag misfitting items, i.e., items which do not contribute to the measurement of a single trait. The criteria set by Wright and Linacre (1994) [30] for infit and outfit for rating scale data are 0.60 to 1.40. Table 2 shows that item 25 is the only misfitting item. In all four models, item 25 was misfitting. Item 25 is *While I am taking tests, I have to go to bathroom*. Figure 1 is the map of the latent distribution and item parameter estimates. The map shows that the numbers of easy items have to be increased to cover the lower part of the scale.

### 4.3. Consecutive Analysis

Consecutive analysis was run to evaluate the unidimensionality of the individual subscales. In this analysis three separate unidimensional models for each subscale was estimated to examine the psychometric characteristics for the subscales.

As Table 3 shows, thresholds are ordered in each subscale and the distance between them in each subscale is satisfactory. The reliability of Thoughts, Off-task Behavior and Autonomic Reactions subscales are 0.82, 0.65, 0.64, respectively. The values of reliability for the Off-Task Behaviors and Autonomic Reactions subscales are low because of the small number of items in these two scales. Off-Task Behaviors subscale has eight items and Autonomic Reactions contains just 9 items while the Thoughts includes 13 items. Principal component analysis of standardized residuals was run to check the unidimensionality of each subscale. The eigenvalues of the first contrasts for each dimension were less than the minimum level of 2. They were equal to 1.8, 1.5 and 1.5 eigenvalue units in Thoughts, Off-Task Behavior and Autonomic Reactions subscales, respectively. Therefore, we can conclude that each subscale is unidimensional and measures only one construct. Findings show that there were not any misfitting items in Thoughts and Off-Task Behaviors subscales, but item 25, in Autonomic Reaction subscale with an infit mean square value of 1.60 and an outfit mean square value of 1.41 was flagged as a misfitting item.

Table 4 shows the latent correlations and the consecutive analysis correlations between the three latent traits. The latent correlations are computed via multidimensional Rasch model. The moderate correlations indicate that the dimensions are separate and each subscale measures a distinct dimension of test anxiety in children.

### 4.4. Raw Score to Interval Measure Conversion

**Figure 2:**
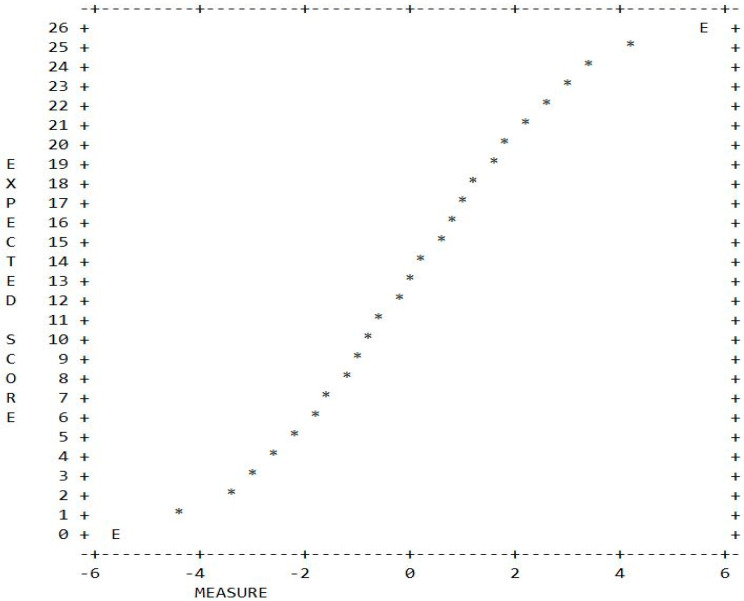
Raw score-Rasch measure conversion plot for the Thoughts scale.

Figure 2 shows the raw score to Rasch measure conversion plot for the Thoughts subscale. That is, for any raw score on the Thoughts subscale (*y* axis) the equivalent Rasch measure can be read from the *x* axis. As the plot shows, the relationship between the raw scores and Rasch measures, which are on an interval scale, is non-linear. At the two ends of the raw score scale, equal distances between scores translate to a bigger difference in the Rasch scale than in the middle of the scale. For example, consider three pairs of respondents with raw scores of 4/5, 13/14, and 24/25. The three pairs are one unit apart on the raw score scale. However, their Rasch measures translate to −2.52/−2.16, 0.00/0.25 and 3.45/4.26, respectively. The three pairs are different by 0.36, 0.25, and 0.81 logit. This is a clear indication that the raw score scale is not interval. 

### 4.5. External Validity

To provide further validity evidence for the PTASC its correlation with an external criterion was examined (Table 5). The magnitude of correlation coefficients reveal that test anxiety as measured by the PTASC is moderately associated with the construct of general and specific anxiety disorders in children. 

## 5. Discussion 

In this study the Children’s Test Anxiety Scale (Wren and Benson, 2004 [4]) was translated into Persian and validated. This particular scale was selected for translation because it has purportedly been designed for multiethnic populations and, therefore, seemed to be a suitable candidate for translation and application in a different language and culture. Evidence supporting the clarity and coherence of the translation was provided by bilingual psychologists and translation experts. The Rasch rating scale model (Andrich, 1978 [16]) was chosen as the measurement model for the analyses. 

Rating scale diagnostics indicated that the rating scale category thresholds 2 and 3 were disordered. This could indicate that children cannot distinguish between *most of the time* and *almost always*. In the next step we collapsed categories 2 and 3 and reduced the number of response options from four to three. This rating scale structure produced the best results in terms of thresholds order and distance as well as fit. Further analyses were performed on this final rating scale structure with three categories.

Principal component analysis of standardized residuals (Smith, 2002 [26]) was applied to assess unidimensionality. PCA of the residuals indicated that the data are not unidimensional. This was not unexpected as the original scale in English had been designed to measure three distinct but correlated dimensions of exam anxiety, namely, *Thoughts, Off-Task Behavior,* and *Autonomic Reactions*. To accommodate separate dimensions of anxiety, multidimensional Rasch rating scale model (Adams, Wilson, and Wang, 1997 [28]) was employed. Likelihood ratio test demonstrated that the three-dimensional model fits the data significantly better than the unidimensional model. This was interpreted as the justifiability of assuming a three-dimensional scale. In other words, computing a total raw score on the scale is not warranted and three separate scores for the three dimensions of test anxiety should be reported. 

In the consecutive analysis, the PCA of residuals confirmed the unidimensionality of each subscale with eigenvalues below 2. The latent correlations from the multidimensional analysis and the correlations from the consecutive Rasch analysis suggest that the construct of exam anxiety in children as measured by the Persian version of the Children’s Test Anxiety Scale is multidimensional, albeit the dimensions are highly correlated. 

Findings revealed that the reliabilities of the subscales are satisfactory in the multidimensional analysis since information is borrowed across the dimensions (Baghaei, 2012/2013 [31,32]). However, in the consecutive analysis the reliabilities of Off-Task Behavior and Autonomic Reactions subscales are relatively low due to the small number of items.

Item fit values indicated that item 25 (I have to go to the bathroom) misfits in all models and should be deleted. Therefore, for the PCTAS we propose a three-dimensional 29-item scale with a three-point Likert scale structure. The misfit of item 25 may suggest that the need to use the bathroom is irrelevant to the construct of exam anxiety. Interestingly, similar findings have also been reported by researchers in the field of health measurement. In instruments designed to measure patients’ physical disability, as a result of stroke or accident, before and after treatment usually ‘bowel’ and ‘bladder’ items misfit (Tennant and Geddes, 1996 [33]; van Hartingsveld, Lucas, Kwakkel, Lindeboom, 2006 [34]). The misfit of these items in the disability scales and in the present instrument could be due to social phobia associated with using the bathroom (Knowles and Skues, 2016 [35]). 

External validation indicated that the PCTAS and its subscales moderately correlate with the subscales of Spence Children’s Anxiety Scale (Spence, 1998 [14]) and its total score, contributing to the validity evidence of the PCTAS. Coefficients of correlation indicated that there are medium correlations between most SCAS scales and PCTAS scales. The correlation between total PCTAS and total SCAS is 0.58 (*n* = 160, *p* < 0.01), while the correlation between the Generalized Anxiety Disorder subscale of the SCAS and the combined PCTAS is 0.60 (*n* = 160, *p* < 0.01). The Physical Injury Fears subscale of the SCAS has the smallest correlations (between 0.12–0.17) with the PCTAS and its subscales. This subscale contains items pertaining to fear of darkness, fear of dogs, fear of doctors, fear of heights, and fear of insects which seem to be irrelevant to test anxiety. These correlations are considered as evidence of convergent and discriminant validity for the scale. The observed patterns of correlations indicate that the PCTAS is correlated with the relevant construct of general and specific anxieties in children while being a distinct construct. 

The present study showed that, excluding one item, the subscales of PCTAS are Rasch scalable. This has several implications for the application of the scale in general and clinical settings. The Rasch model is the only item response theory model which satisfies the requirements of conjoint measurement which is necessary to create an interval scale (Van Newby, Conner, and Bunderson, 2009 [36]). If the measurement of the effects of intervention or change over time is required, measures on an interval scale should be used as it has a unit which maintains its size across the whole parts of the scale. The fit of data to the Rasch model also indicates that the construct of interest is quantitative and the total raw score can be used as an indicator of the respondents‘ latent trait (Wright, 1989 [37]). 

## 6. Conclusions

Findings of this study revealed that the Persian translation of the CTAS is valid and reliable for measuring exam anxiety in Iranian students. Further research should be conducted on the applicability of the PCTAS in other Persian-speaking countries. One limitation of the study is that differential item funning (DIF) for the items across subpopulations was not investigated. Due to the relatively small sample size in this study, partitioning respondents to smaller subgroups for DIF analysis would not have yielded reliable estimates. It is interesting and of great significance to examine whether the items work differently or are biased against certain age groups, cultural minorities and males or females. Examination of the latent classes of respondents who might interpret the items differently or respond in peculiar ways is another potential line of research.

## Figures and Tables

**Table 1:** Characteristics of the models and scoring procedures.

Model Scoring	Thresholds	G²	Reliability	No. Parameters	Misfitting Items	AIC	CAIC	BIC
1-dim 0123	−0.39 0.35 −0.04	9543.13	0.95	33	22, 25	9609.13	9743.44	9710.44
1-dim 0122	−0.18 0.18	8028.20	0.94	32	22, 25	8092.20	8222.44	8190.44
1-dim 0112	−1.01 1.01	7439.54	0.93	32	25	7503.54	7633.78	7601.78
3-dim 0112	−1.15 1.15	7307.87	0.87 0.86 0.85	37	25	7381.87	7532.46	7495.46

Note: G^2^ = −2 loglikelihood; AIC = Akaike Information Criterion; CAIC = Consistent Akaike.

**Table 2:** Item parameters and fit statistics in the three-dimensional model.

Item	Estimate	Error	Infit MNSQ	Outfit MNSQ
1	−0.733	0.101	0.86	0.88
2	−1.446	0.109	0.97	0.99
3	−0.274	0.105	0.81	0.79
4	0.214	0.120	1.07	1.04
5	0.125	0.104	0.81	0.79
6	0.367	0.106	0.85	0.85
7	0.471	0.106	0.97	0.92
8	0.585	0.124	1.23	1.14
9	1.107	0.111	1.08	0.97
10	0.396	0.122	1.15	1.02
11	0.424	0.106	1.05	1.04
12	−0.233	0.105	1.05	1.13
13	−1.422	0.102	1.04	1.05
14	0.190	0.107	1.25	1.17
15	−0.781	0.102	0.65	0.64
16	−0.268	0.102	1.04	1.04
17	0.615	0.124	0.91	0.76
18	0.168	0.106	0.98	0.90
19	0.548	0.107	0.90	0.81
20	−0.708	0.113	1.24	1.32
21	−0.435	0.102	0.82	0.84
22	0.028	0.106	1.35	1.38
23	0.102	0.119	1.10	0.92
24	0.186	0.104	0.89	0.81
25	0.542	0.124	1.67	1.55
26	−0.904	0.103	0.99	0.98
27	0.653	0.108	0.72	0.68
28	−0.300	0.338	0.81	0.67
29	0.229	0.362	1.23	1.13
30	0.553	0.280	1.30	1.25

**Table 3:** Scale properties in the consecutive analysis.

	Thresholds	Reliability	Eigenvalue (First Contrast)	Misfitting Items	Variance
Thoughts	−1.52 1.52	0.82	1.8	-	3.22
Off-task	−1.14 1.14	0.65	1.5	-	2.54
Autonomic	−1.11 1.11	0.64	1.5	25	2.97

**Table 4:** Correlations between the dimensions.

	1	2	3
Thoughts	-	0.62	0.62
Off-task	0.73	-	0.56
Autonomic	0.79	0.72	-
Variance	1.92	1.67	2.51

**Table 5:** Correlations between SCAS subscales and PTASC subscales.

Scales	1	2	3	4	5	6	7	8	9	10	11
Thoughts	0.89										
Off-Task	0.58 **	0.79									
Autonomic	0.69 **	0.57 **	0.83								
PTASC	0.91 **	0.80 **	0.85 **	0.92							
Separation	0.26 **	0.19 *	0.24 **	0.27 **	0.65						
Social	0.53 **	0.40 **	0.42 **	0.53 **	0.41 **	0.62					
Obsessive	0.45 **	0.36 **	0.39 **	0.47 **	0.42 **	0.38 **	0.60				
Panic	0.38 **	0.24 **	0.45 **	0.42 **	0.43 **	0.31 **	0.45 **	0.73			
Physical	0.17 *	0.12	0.12	0.16 *	0.47 **	0.31 **	0.19 *	0.24 **	0.57		
Generalized	0.54 **	0.42 **	0.59 **	0.60 **	0.47 **	0.55 **	0.52 **	0.50 **	0.39 **	0.60	
SCAS	0.55 **	0.41 **	0.53 **	0.58 **	0.75 **	0.69 **	0.70 **	0.70 **	0.60 **	0.81 **	0.81

Note: Cronbach’s alphas on the diagonal; * indicates *p* < 0.05; ** indicates *p* < 0.01; PTASC: the sum score for the Persian Test Anxiety Scale for Children; SCAS: the sum score for the Spence Children’s Anxiety Scale
